# Supplementary material for: On the Prediction of In Vitro Arginine Glycation of Short Peptides Using Artificial Neural Networks
Source: Sensors (Basel). 2022 Jul 13;22(14):5237. doi: 10.3390/s22145237 (PMC9324327; doi:10.3390/s22145237)
Supplement: Supplementary file 1 [file sensors-22-05237-s001.zip › Supplementary material - SI and Figure S1.pdf]

sensors

ISSN 1424-8220
www.mdpi.com/journal/sensors

Supplementary information

On the prediction of *in vitro* arginine glycation of short peptides using artificial neural networks

Ulices Que-Salinas[1], Dulce Martinez-Peon[2], Angel D. Reyes-Figueroa[3,4], Ivonne Ibarra[5], Christian Quintus Scheckhuber[6,] *

1 Centro de Ciencias de la Tierra, Universidad Veracruzana, Xalapa 91090, VER, Mexico; feanorkdt@gmail.com
2 Department of Electrical and Electronic Engineering, National Technological Institute of Mexico/IT, Monterrey 67170, NL, Mexico; dulce.mp@nuevoleon.tecnm.mx
3 Consejo Nacional de Ciencia y Tecnología, Av. Insurgentes Sur 1582, Col. Crédito Constructor, Benito Juárez, Mexico City 03940, DF, Mexico; angel.reyes@cimat.mx
4 Centro de Investigación en Matemáticas Unidad Monterrey, Parque de Investigación e Innovación Tecnológica (PIIT), Av. Alianza Centro No. 502, Apodaca 66628, NL, Mexico
5 Independent Researcher, Monterrey 66620, NL, Mexico; livonneibarra@gmail.com
6 Departamento de Bioingeniería, Escuela de Ingeniería y Ciencias, Tecnologico de Monterrey, Ave. Eugenio Garza Sada 2501, Monterrey 64849, NL, Mexico
* Correspondence: c.scheckhuber@tec.mx

1.1 Table of properties

For the amino acid properties, we included eight features: amino acid sequence of the peptide (SoA), hydropathy (Hyd)[1], mass (Mas)[2], hydrophobicity (Hyp)[3], polarizability (Pol)[4], normalized van der Waals volume (vdW)[5], torsion angle (ToA)[6], and isoelectric point (IEP)[7], see Tab. S1. To generate the SoA feature we arranged in descending order the amino acids by the hydropathy index. We set the 0 to the closest number (Glycine) and start an increasing/decreasing numbering using only integers. If they should have the same value, we sorted them by mass.

1.2 Table of vectors

For the table of created vectors see Tab. S2.

1.3 Artificial neural network operation (ANN)

To illustrate an ANN operation, consider the training process related to the MLP employed for the case considering hydropathy of the peptide's amino acids (case IB). For this we have 11 input neurons (one neuron for each amino acid), tree hidden layers with 4, 3 and 2 neurons, and only one output neurons for the estimation of the probability of glycation "G", where the relation between an input vector $\hat{P}$ (that represent all the patterns in the training set) and the G_k output value is determined by the expression:

$$G_k = C\left(\tilde{b}_k + \sum_{l=1}^{20} \tilde{w}_{lk} \ * B\left(\tilde{b}_l + \sum_{j=1}^{40} \tilde{w}_{jl} * A\left(\sum_{i=1}^{11} w_{ij} * \hat{P} + b_j\right)\right)\right) \qquad (S1)$$

where w_{ij}, $\tilde{w}_{jl}$ and $\tilde{w}_{lk}$ are the connection weights between the neurons in input-hidden, hidden-hidden and hidden-output layers, respectively. The weights allow the perceptron to evaluate the relative importance of each of the outlets[8]. Neural network algorithms learn by discovering better and better weights that result in a more accurate prediction. There are several algorithms that are used to fine tune the weights, the most common is called backpropagation, which is used for all cases in this work. b_j, $\tilde{b}_l$ and $\tilde{b}_k$ are some extra weights called bias operating as thresholds. These weights allow you to move the curve of the activation function up and down or left and right on the number graph. This enables the numerical output of the perception to be adjusted. A, B and C are the ReLu activation functions between each consecutive layer.

To ensure that an ANN gives satisfactory results, its weights must be adjusted. The way to do this is by implementing a supervised training algorithm, that consists of introducing 38 examples into the ANN in which their training can be oriented in the right direction. Each example consists in *p* inputs denoted by $\hat{P}_{\mathrm{p}}$ and *k* outputs denoted by $\hat{G}_k{}^p$, where the index $p = 1$,…,38 represents the number

of patterns used in the network training process. Thus, the task now is to find a way to minimize the error between the estimations ${G_k}^p$ made by the network (the outputs) and the corresponding values ${\hat{G}_k}^p$ expected to be predicted by the ANN during the training stage. This is done by a minimization of the weight dependent error function $E(\vec{w})$ (also called cost function)[9], which has several expressions in the literature. For this work, we consider one of the most recurrent expressions for the error function, defined for case I as

$$E(\vec{w}) = \frac{1}{38}\sum_{p=1}^{38}\sum_{k=1}^{1}\frac{1}{2}\left({G_k}^p - {\hat{G}_k}^p\right)^2 \qquad (S2)$$

The minimization of the error function is made by the iteration of the Adam algorithm, known as *backpropagation*[10,11], which searches the direction in which the error decreases the most on each epoch. One epoch means that each one of the 38 examples in the training sample of the data set has had an opportunity to update the internal model parameters (the weights). This search is done by changing the value for each w_{ij} in the next epoch $t_e + 1$, propagating the error at epoch t_e from output to input neurons by the rule

$$w_{ij}(t_e + 1) = w_{ij}(t_e) - \gamma\frac{\partial E(t_e, \vec{w})}{\partial w_{ij}(t_e)} + \alpha \bigtriangleup w_{ij}(t_e) \qquad (S3)$$

with $0 < \gamma < 1$ called the *learning rate*[12], $\alpha \bigtriangleup w_{ij}(t_e)$ is called the momentum term, added for preventing getting trapped in a local minimum, and $0 < \alpha < 1$ another parameter to be adjusted. The number of epochs for the training process is a hyperparameter that defines the number times that the learning algorithm will work through the entire training database. This whole process is carried out for the training of each of the cases studied, considering that due to the small size of the database, the results presented below for each case were averaged over the predictions made by

100 different ANNs already trained. It is worth mentioning that for all architectures of this study, we have used a rectified linear unit (ReLu) as an activation function for the layers[12-14].

The specific architecture of the ANNs used for each case is presented in Table 1 of the main manuscript. Regarding the number of epochs, this value was adjusted for each case according to the time when, epoch by epoch, the improvement in accuracy was negligible compared to the number of epochs invested for such improvement. This is measured by considering the value of the cost function applied to the validation set at the end of each epoch, considering that the network has not been trained with it at all. This not only allows to stop the learning process when a certain desired accuracy has been reached, but also allows to monitor the process itself and to ensure that the ANN does not start to overtrain; that is, that the error does not start to increase for the validation set as it decreases for the training set. Finally, for each one of the study cases we have developed a database to train and test the ANN, selecting in general around 15% of the data of each database for the corresponding test set, and another 15% for the validation set. Considering the small number of proteins available to form the databases, a series of different distributions of the data sets was performed to ensure that the results were consistent as they were not conditioned to a specific distribution of the proteins in the training, validation, and prediction sets.

1.4 Results for case 2

The following figures shows the results of MAE and MAPE for the 28 property combinations of case 2.

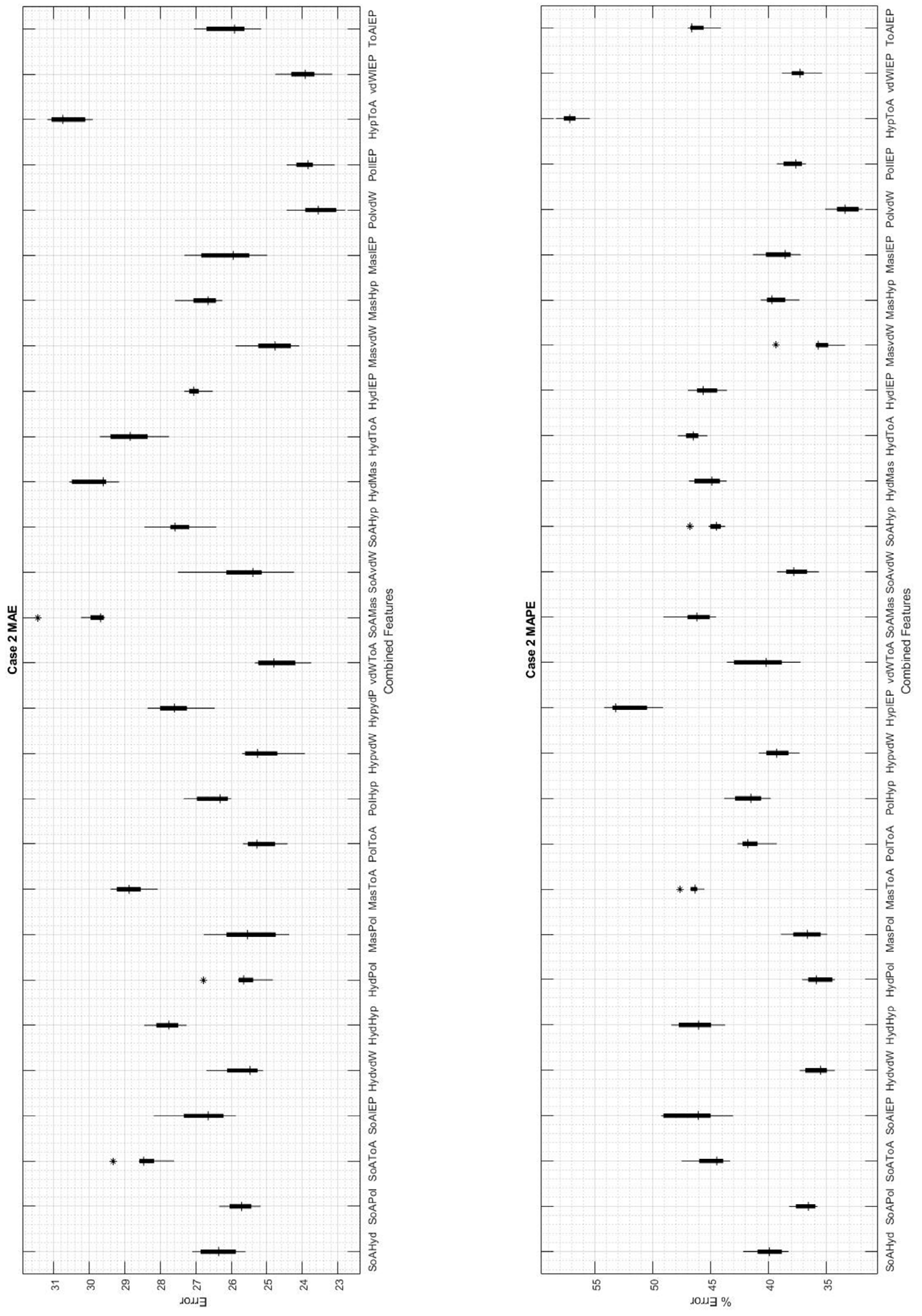

(a) **(b)**

Figure S1. Box plot of the results for case 2 for all the combinations of case 1, a) shows the values obtained for MAE, b) shows the values obtained for MAPE. The * sign denotes outliers.

Reference list for supplementary material

1. Markus G, Tritsch GL, Parthasarathy, R. A model for hydropathy-based peptide interactions. *Archives of Biochemistry and Biophysics* 1989; 272: 433-439.
2. Chiavari G, Galletti GC. Pyrolysis—gas chromatography/mass spectrometry of amino acids. *Journal of Analytical and Applied Pyrolysis* 1992; 24 (2): 123-137.
3. Fauchère JL, Charton M, Kier LB, Verloop A, Pliska V. Amino acid side chain parameters for correlation studies in biology and pharmacology. *Int J Pept Protein Res* 1988; 32(4): 269-78. doi: 10.1111/j.1399-3011.1988.tb01261.x. PMID: 3209351.
4. Marie-Paule Lefranc. (2001). Amino acids. *Accessed on* 07/06/2022, https://www.imgt.org/IMGTeducation/Aide-memoire/_UK/aminoacids/
5. Bachem. (2017). Peptide calculator. *Accessed on* 07/06/2022, https://www.bachem.com/knowledge-center/peptide-calculator/
6. Sjoblom NM, Kelsey MMG, Scheck RA. A Systematic Study of Selective Protein Glycation. *Angew Chemie - Int Ed* 2018; 57: 16077–16082.
7. Jha, K, Saha, S, Tanveer, M. Prediction of protein-protein interactions using stacked auto-encoder. *Trans Emerging Tel Tech.* 2021; e4256. https://doi.org/10.1002/ett.4256R .
8. F. Rosenblatt, Principles of Neurodynamics: Perceptrons and the Theory of Brain Mechanisms (Spartan Books, Washington, 1962).
9. Rojas, Neural Networks: A Systematic Introduction. *Springer-Verlag* 1996.
10. Rumelhart DE, Hinton GE, Williams RJ. Learning representations by back-propagating errors. *Nature* 1986; 323: 533–536.
11. Bishop C. Machine Learning for Pattern Recognition. *Springer- Verlag* 2006.
12. Goodfellow, I. *et al.* A. Deep Learning. *The MIT Press* 2016.
13. Ketkar, N. *Deep Learning with Python: A Hands-on Introduction* 2017.
14. K. Eckle and J. Schmidt-Hieber, A comparison of deep networks with ReLU activation function and linear spline-type methods. *Neural Networks* 2019; 110: 232-242.
